# Accuracy of cobas MTB and MTB-RIF/INH for Detection of *Mycobacterium tuberculosis* and Drug Resistance

**DOI:** 10.1016/j.jmoldx.2024.05.004

**Published:** 2024-08

**Authors:** Margaretha de Vos, Anura David, Karthickeyan Duraisamy, Darshaalini Nadarajan, Ecaterina Noroc, Adam Penn-Nicholson, Valeriu Crudu, Sidhartha Giri, Florian P. Maurer, Sanghamitra Pati, Wendy Stevens, Lesley Scott, Jyotimayee Turuk, Samuel G. Schumacher, Morten Ruhwald, Study Group, Nelly Ciobanu, Sarabijt Singh Chadha, Alexandru Codreanu, Dasarathi Das, Trish Kahamba, Itishree Kar, Aurélien Macé, Jyoti Mohanty, Pamela Nabeta, Katherina Kranzer, Stefano Ongarello, Archana Pattanaik, Elena Romancenco, Sanjay Sarin, Lyndel Singh, Sunita Singh, Nadia Turcan

**Affiliations:** ∗ FIND, Geneva, Switzerland; † Wits Diagnostic Innovation Hub, Health Sciences Research Unit, Faculty of Health Sciences, University of Witwatersrand, Johannesburg, South Africa; ‡ FIND, New Delhi, India; § National and WHO Supranational Reference Center for Mycobacteria, Research Center Borstel, Borstel, Germany; ¶ Phtisipneumology Institute "Chiril Draganiuc," Chisinau, Republic of Moldova; ‖ Indian Council of Medical Research–Regional Medical Research Centre, Bhubaneswar, India; ∗∗ Institute of Medical Microbiology, Virology and Hospital Hygiene, University Medical Center Hamburg-Eppendorf, Hamburg, Germany; †† The National Priority Program of the National Health Laboratory Service, Johannesburg, South Africa

## Abstract

This study evaluated the performance of cobas MTB and cobas MTB-RIF/INH for the diagnosis of tuberculosis and detection of rifampicin (RIF) and isoniazid (INH) resistance. Adults presenting with pulmonary tuberculosis symptoms were recruited in South Africa, Moldova, and India. Performance of cobas MTB was assessed against culture, whereas cobas MTB-RIF/INH was assessed using phenotypic drug susceptibility testing and whole-genome sequencing as composite reference standards. Xpert MTB/RIF (Xpert) or Xpert MTB/RIF Ultra (Ultra) was used as a comparator. The overall sensitivity and specificity of cobas MTB were 95% (95% CI, 93%–96%) and 96% (95% CI, 95%–97%). Among smear-negatives, the sensitivity of cobas MTB was 75% (95% CI, 66%–83%). Among participants tested with both cobas MTB and Xpert, sensitivity was 96% (95% CI, 94%–97%) for cobas MTB and 95% (95% CI, 93%–97%) for Xpert. Among participants tested with both cobas MTB and Ultra, sensitivity was 88% (95% CI, 81%–92%) for cobas MTB and 89% (95% CI, 83%–93%) for Ultra. Sensitivity and specificity of cobas MTB-RIF/INH for RIF and INH detection were 90% (95% CI, 84%–94%) and 100% (95% CI, 99%–100%), and 89% (95% CI, 84%–93%) and 99.5% (95% CI, 98%–100%), respectively. The cobas MTB and cobas MTB-RIF/INH assays exhibited high performance in a diverse population and present a suitable option for molecular detection of tuberculosis and RIF and INH resistance.

Drug-resistant tuberculosis (TB) continues to threaten global TB control.^1^ From 2018 to 2022, 825,000 individuals globally with rifampicin (RIF)–resistant or multi–drug-resistant TB [resistant to at least RIF and isoniazid (INH)] have been reported to have initiated treatment, which is only 55% of the World Health Organization (WHO) target of 1.5 million for the period.^1^ Delays or failures in the detection of drug-resistant TB lead to increased risk of patient mismanagement, drug resistance amplification, and ongoing disease transmission.^2^ Improving accuracy and speed of diagnosis is key to improving patient outcomes and preventing spread of TB and drug-resistant TB.

In 2021, the WHO endorsed moderate complexity nucleic acid amplification tests for initial diagnosis of TB and detection of RIF and INH resistance.^3^ Tests included in this class are largely fully automated and less complex than conventional culture-based methods [such as phenotypic drug susceptibility testing (pDST) and whole-genome sequencing (WGS)] and offer potential for rapid high-throughput testing. With the addition of INH susceptibility detection, they overcome the limitations of Xpert MTB/RIF (Xpert; Cepheid, Sunnyvale, CA) and Xpert MTB/RIF Ultra (Ultra; Cepheid), which only detect RIF susceptibility.

The cobas MTB and MTB-RIF/INH assays (Roche Diagnostics International AG, Rotkreuz, Switzerland) have been endorsed by WHO in the moderate complexity nucleic acid amplification test class.^3^ cobas MTB is an automated, qualitative nucleic acid amplification test for *Mycobacterium tuberculosis* complex (MTBC) detection from raw, digested, or decontaminated sputum and bronchoalveolar lavage samples. cobas MTB-RIF/INH is a reflex assay for the detection of RIF resistance-associated mutations in *rpoB* and INH resistance-associated mutations in *katG* and the *inhA* promoter region. Both cobas assays are performed on the cobas 6800 or 8800 closed platforms. In previous studies, cobas MTB exhibited high sensitivity against a culture reference standard,^4^^,^^5^ and performance of cobas MTB-RIF/INH was sufficient for WHO endorsement.^5^ However, further evaluation of these assays in geographically diverse settings is required to inform global and national policy decision-making. The objective of the current study was to evaluate the clinical diagnostic accuracy of cobas MTB and cobas MTB-RIF/INH in settings of intended use, including those with a high prevalence of individuals living with HIV.

## Materials and Methods

### Study Design

This multicenter, cross-sectional, clinical diagnostic accuracy study evaluated the performance of cobas MTB for MTBC detection and cobas MTB-RIF/INH for RIF and INH resistance detection (NCT04147676; *https://www.clinicaltrials.gov*, last accessed March 14, 2024). Xpert and Ultra were included in the evaluation as comparator tests as both assays are WHO endorsed and implemented for routine patient care in their intended setting. Xpert was used in Moldova and India, whereas Ultra was used in South Africa. The reference standard for MTBC detection was liquid culture confirmed by a WHO-recommended rapid or molecular test. The reference standard for RIF and INH resistance was a composite of pDST and WGS. The study was approved by relevant institutional review boards and independent ethics committees. All participants provided written informed consent.

### Participants

Participants were prospectively recruited sequentially between May 17, 2019, and November 30, 2021 at three sites: University of the Witwatersrand (Johannesburg, South Africa); Phthisiopneumology Institute “Chiril Draganiuc” (Chisinau, Moldova); and Indian Council of Medical Research–Regional Medical Research Center (Bhubaneswar, India).

Adults (≥18 years of age) presenting with symptoms or characteristics consistent with pulmonary TB, in outpatient clinic or inpatient hospital settings, were eligible for inclusion. Criteria indicating clinical suspicion of pulmonary TB were cough of ≥2 weeks’ duration (≥1 week for HIV co-infected individuals in South Africa) and one or more of the following: fever, malaise, recent weight loss, night sweats, hemoptysis, chest pain, loss of appetite, or contact with an individual with active TB. Participants were required to provide sputum samples of adequate volume (two ≥3-mL samples in Moldova and India, and four ≥2-mL samples in South Africa) over one or two visits, depending on the site protocol. Receipt of any dose of TB treatment before 6 months of enrollment resulted in exclusion.

Retrospectively collected frozen raw sputum samples from the FIND Specimen Bank (*https://www.finddx.org/what-we-do/cross-cutting-workstreams/biobank-services*, last accessed March 14, 2024) were also used, to achieve accurate performance estimates through a diverse collection of resistance-conferring mutations. To that end, a random selection of samples that tested positive for RIF- or INH-resistant TB in previous phenotypic and genotypic investigations was included in the study.

### Sample Processing and Analytical Workup

**Figure 1:**
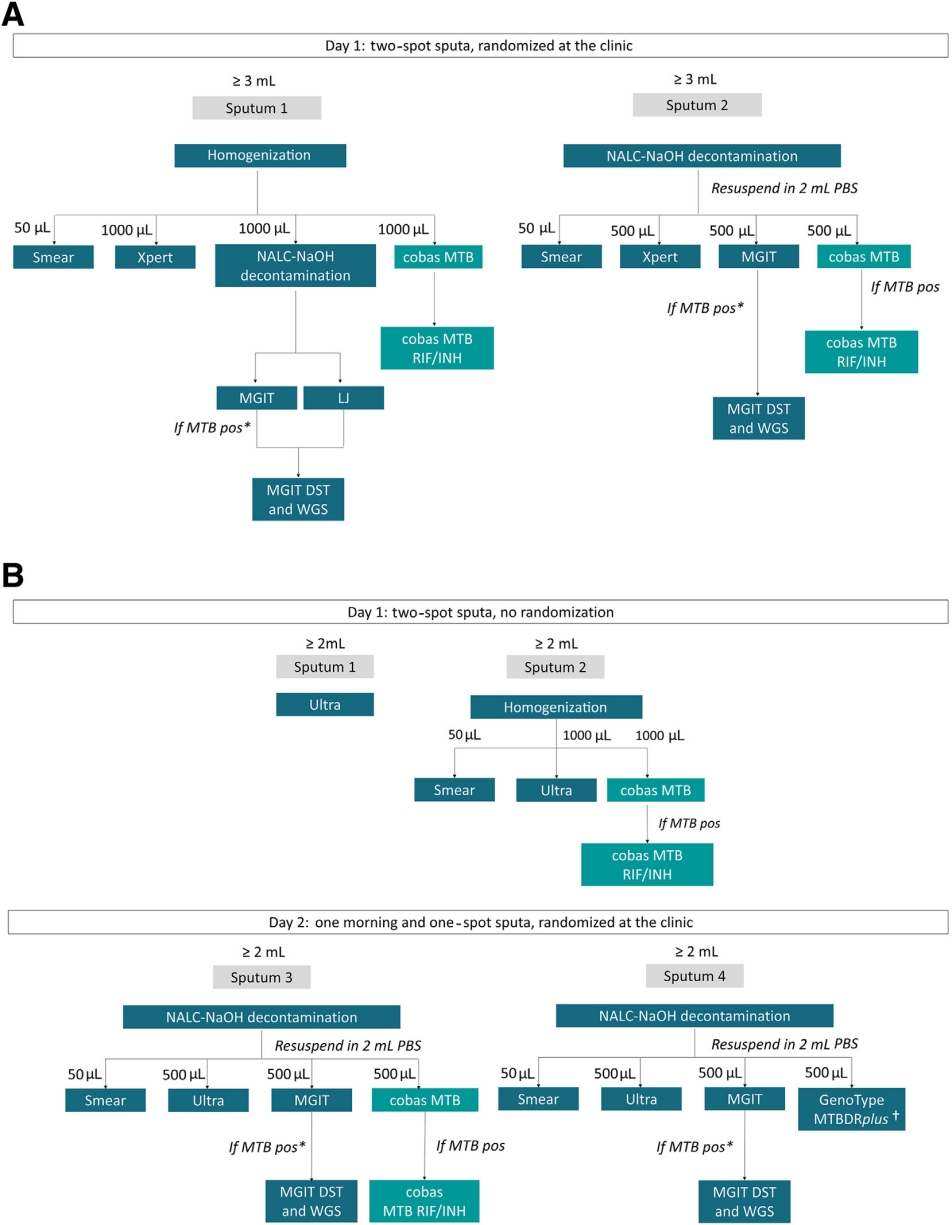
Study flow for samples provided by participants from Moldova and India (**A**) and South Africa (**B**). In Moldova and India, eligible participants were asked to provide two sputum specimens on the same day. The sputum specimens were randomized for testing, where direct cobas and Xpert testing was done on sputum 1, and indirect [after N-acetyl-l-cysteine/sodium hydroxide (NALC-NaOH) decontamination] cobas and Xpert testing was done on sputum 2. In South Africa, eligible participants were asked to provide four sputum specimens on 2 separate days. The two sputum specimens collected on day 1 were not randomized; sputum 1 was used for Ultra testing as per national standard of care, whereas sputum 2 was used for direct Ultra and cobas testing. No culture was done on day 1. Sputum specimens collected on day 2 were randomized for testing, where indirect (after NALC-NaOH decontamination) cobas and Ultra testing were done on sputum 3. ∗Testing was only done if *Mycobacterium tuberculosis* complex confirmed positive (pos) from BACTEC Mycobacteria Growth Indicator Tube 960 (MGIT) or Löwenstein-Jensen (LJ). †GenoType MTBDR*plus* was only done on smear-positive NALC-NaoH decontaminated pellets. DST, drug susceptibility testing; PBS, phosphate-buffered saline; WGS, whole-genome sequencing.

Evaluations were performed at reference laboratories at the recruitment sites in South Africa and India. Samples from Moldova were delivered weekly to the WHO Supranational Reference Center for Mycobacteria (Borstel, Germany), for testing. In India and Germany, the two samples from each participant were randomized on arrival at the laboratory. The first sample was homogenized and used for direct cobas MTB, cobas MTB-RIF/INH, and Xpert testing, acid-fast bacilli smear microscopy, and N-acetyl-l-cysteine/sodium hydroxide decontamination before BACTEC Mycobacteria Growth Indicator Tube 960 (Becton Dickinson, Franklin Lakes, NJ) (MGIT; liquid) or Löwenstein-Jensen (solid) culture. The second sample was N-acetyl-l-cysteine/sodium hydroxide decontaminated before all the aforementioned tests (Figure 1A). In South Africa, the two samples collected on day 1 were not randomized (Figure 1B). Sample 1 was tested with Ultra per national standard of care, whereas sample 2 was homogenized for direct cobas MTB, Ultra, and smear testing. Samples 3 and 4, collected on day 2, were randomized and decontaminated before all testing. Both samples 3 and 4 were used for smear microscopy and MGIT; sample 3 was also used for cobas MTB and Ultra testing, and sample 4 was used for GenoType MTBDR*plus* (Bruker Hain Lifescience, Nehren, Germany) molecular testing per national standard of care. At all sites, cobas MTB RIF/INH was performed as a reflex test on all cobas MTB–positive samples.

To confirm MTBC positivity, all positive cultures were further tested with either the Bioline TB Ag MPT64 assay (Abbott Laboratories, Chicago, IL) or GenoType MTBDR*plus*. pDST was performed using the MGIT960 SIRE kit (Becton Dickinson) with cutoffs of 1 and 0.1 μg/mL for RIF and INH, respectively. WGS was performed at Medgenome (Bangalore, India), by means of DNA extracted using an in-house heat inactivation/bead beating method, and DNA was extracted using the cetyltrimethylammonium bromide method from the laboratories in Germany and South Africa. Mutations in the *rpoB*, *katG*, *inhA* promoter, *oxyR-ahpC* intergenic region, and *fabG1* genes were recorded and classified per the WHO mutations catalog, second edition.^6^ Clinical information and reference standard results were blinded to the operators of the investigational product, and vice versa. If sufficient sample volume was available, repeat cobas MTB and cobas MTB-RIF/INH testing was performed for the following cases: i) confirmed culture positive and cobas MTB negative; ii) culture negative and cobas MTB positive; iii) pDST susceptible and cobas MTB-RIF/INH resistant (RIF or INH); and iv) pDST resistant and cobas MTB-RIF/INH susceptible (RIF or INH). Repeat pDST was also performed for scenarios iii and iv. Repeat test results were not included in the analyses.

### Outcomes and Statistical Analysis

The primary outcomes were clinical sensitivity and specificity of cobas MTB for MTBC detection, and of cobas MTB-RIF/INH for RIF and INH resistance detection, compared with the reference standards. Point estimates and 95% CIs were calculated using the Wilson score method. Both outcomes were also assessed for Xpert and Ultra, and were evaluated overall and by smear grade. Both sample processing methods were pooled for the purposes of these analyses. Sample 2 from South Africa was excluded from overall analyses for cobas MTB as no reference standard was available but was included in calculations of Ultra performance (using culture results from samples 3 or 4 as the reference standard). Secondary analyses applied the same measures in participant subgroups based on HIV status, recruitment site, TB history, and sample processing method. The number and rate of unsuccessful results for cobas MTB (invalid results) and cobas MTB-RIF/INH (defined as invalid drug resistance within validly detected MTB cases) were also recorded.

Target sample size was 1000 participants, supplemented with an additional 50 RIF-resistant and 50 INH-resistant samples from the FIND Specimen Bank.^7^ The sample size was derived with the assumption of a prevalence of 25% for TB, and 30% for smear-negative TB among all TB, using the formulas described by Zhou et al^8^ in 2011 with power set at 80% and α set at 5%. The intention-to-treat population was defined as all participants who provided written informed consent and who had any test result. The per-protocol population excluded participants who were found to fail eligibility criteria after enrollment, and those with invalid or missing index or reference standard results.

## Results

### Participants

**Figure 2:**
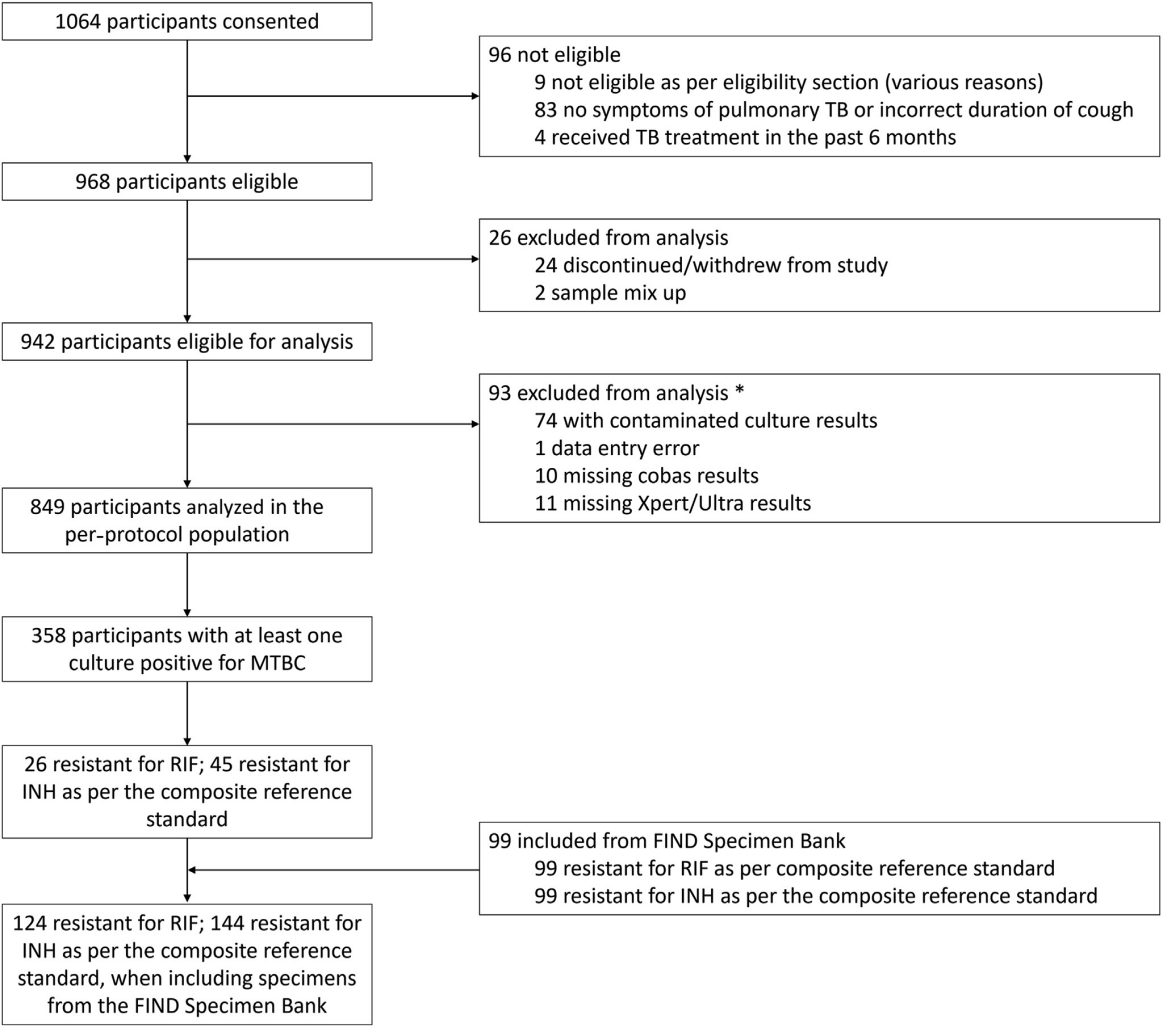
Participant flow. ∗Some reasons for exclusion overlap. INH, isoniazid; MTBC, *Mycobacterium tuberculosis* complex; RIF, rifampicin; TB, tuberculosis.

Of 1064 patients who provided consent, 96 were ineligible for enrollment, 24 withdrew, and 2 were excluded because of sample mix up (Figure 2 and Supplemental Table S1). A further 93 were excluded because of missing results, contaminated culture, or data entry error, leaving 849 participants in the per-protocol population. For RIF and INH resistance detection, a further 99 samples from the FIND Specimen Bank were included.

The median age was 42 years, 30% were female, 19% were HIV positive, and 22% had a history of TB (Table 1). In total, 37% were culture positive and 32% were smear positive. There were considerable differences between the three sites. The site in South Africa had the highest HIV-positivity rate at 55%, compared with 3.7% and 1.5% for Moldova and India, respectively. The site in Moldova had the highest MTBC-positive rate at 69% (the Phthisiopneumology Institute “Chiril Draganiuc” is a national TB referral hospital), with also the highest smear-positive rate at 57%, compared with 19% and 26% for South Africa and India, respectively.

**Table 1:** Demographics and Clinical Characteristics (Per-Protocol Population)

Analyzed population	Total (*N* = 849)	Moldova (*N* = 246)	South Africa (*N* = 274)	India (*N* = 329)
Age, median (range), y	42 (18 to 85)	46 (19 to 81)	38 (18 to 79)	44 (18 to 85)
Female sex, % (*n/N*)	30.3 (257/847)	22.8 (56/246)	35.8 (98/274)	31.5 (103/327)
HIV positive, % (*n/N*)	19.3 (164/849)	3.7 (9/246)	54.7 (150/274)	1.5 (5/329)
History of TB, % (*n/N*)	21.6 (183/849)	22.4 (55/246)	21.5 (59/274)	21.0 (69/329)
Culture positive, % (*n/N*)				
All sample matrices	37.5 (636/1698)	68.5 (337/492)	24.8 (136/548)	24.8 (163/658)
Sputum samples	37.9 (322/849)	68.7 (169/246)	24.6 (68/274)	25.8 (85/329)
Pellet samples	37.0 (314/849)	68.3 (168/246)	20.2 (72/274)	23.7 (78/329)
Smear positive, % (*n/N*)				
All sample matrices	32.5 (551/1697)	56.4 (277/491)	19.0 (104/548)	25.8 (170/658)
Sputum samples	31.0 (263/849)	54.5 (134/491)	18.6 (51/274)	23.7 (78/329)
Pellet samples	34.0 (288/848)	58.4 (143/491)	19.3 (53/274)	28.0 (92/329)
Smear negative and culture positive, % (*n/N*)				
All sample matrices	17.9 (114/635)	18.7 (63/337)	24.3 (33/136)	11.1 (18/162)
Sputum samples	20.9 (67/321)	22.4 (38/169)	25.0 (17/68)	14.2 (12/84)
Pellet samples	14.9 (47/314)	14.9 (25/168)	22.2 (16/72)	7.7 (6/78)
Xpert MTB/RIF or Ultra TB positive, % (*n/N*)		Xpert	Ultra	Xpert
All sample matrices	—	66.5 (327/492)	23.9 (131/548)	28.4 (187/658)
Sputum samples	—	68.7 (169/246)	21.9 (60/274)	26.7 (88/329)
Pellet samples	—	64.2 (158/246)	25.9 (71/274)	30.1 (99/329)
cobas MTB positive, % (*n/N*)				
All sample matrices	35.8 (654/1698)	67.9 (334/492)	23.5 (129/548)	29.0 (191/658)
Sputum samples	38.8 (329/849)	70.3 (173/246)	23.4 (64/274)	28.0 (92/329)
Pellet samples	38.3 (325/849)	65.4 (161/246)	23.7 (65/274)	30.1 (99/329)

–, Not calculated; TB, tuberculosis.

### Diagnostic Accuracy of cobas MTB for the Detection of MTBC

A total of 1698 specimens (both sample types) had valid cobas MTB and culture results. Of these specimens, 636 were culture positive for MTBC, 521 were smear positive and culture positive, and 114 were smear negative and culture positive. Per protocol, all smear-positive MTBC culture-negative samples (*n* = 30 from 24 participants) were excluded, as they may represent an imperfect reference standard, indicate the presence of other mycobacteria, or include samples from participants who received unreported treatment.

**Figure 3:**
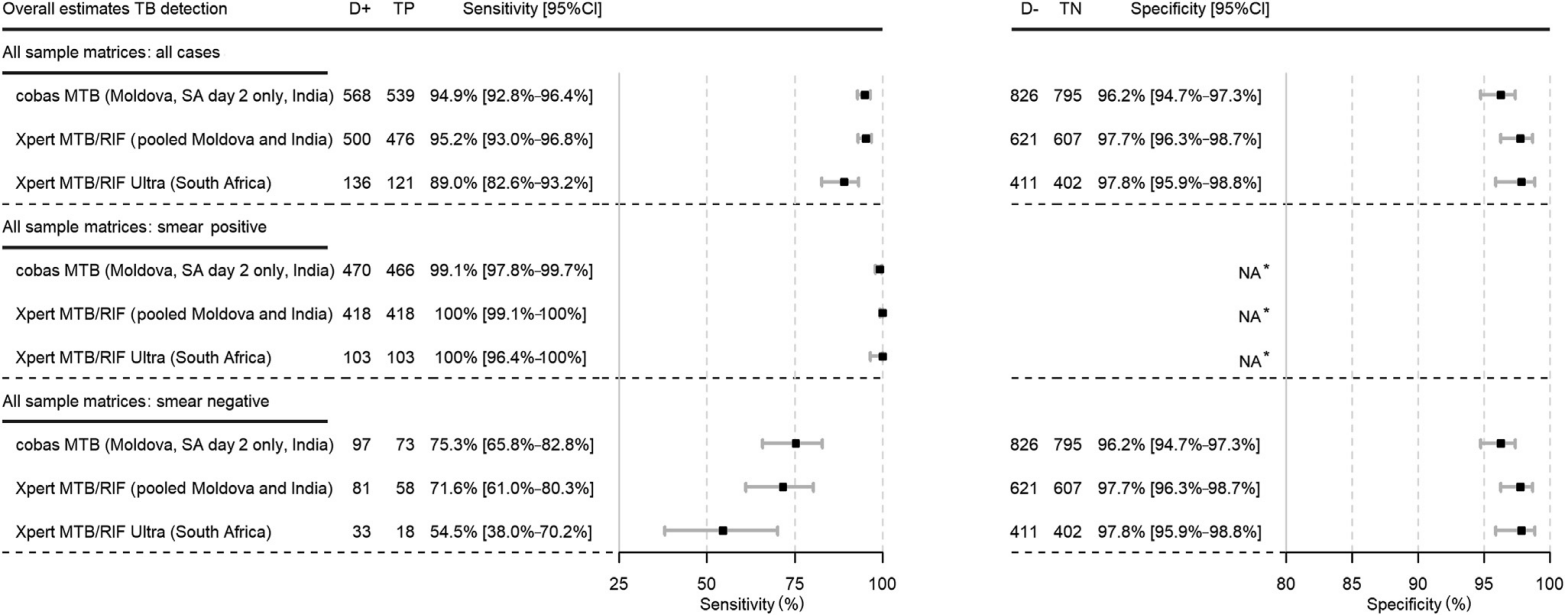
Sensitivity and specificity of cobas MTB, Xpert, and Ultra for *Mycobacterium tuberculosis* complex detection (pooled analysis of both sample processing methods). Overall estimates for cobas MTB include results from both sputa collected in Moldova and India, and only results from the sputum 3 (indirect testing) collected on day 2 in South Africa (SA). Results from sputum 2 (direct testing) collected on day 1 were excluded as no culture reference standard was done on day 1. Overall estimates for Xpert MTB/RIF include results from both sputa collected in Moldova and India. Overall estimates for Xpert MTB/RIF Ultra include results from sputum 2 and 3 collected in South Africa. ∗Smear-positive, culture-negative specimens were excluded from the evaluation. D+, reference standard positive; D−, reference standard negative; NA, not applicable; TB, tuberculosis; TN, true negative; TP, true positive.

In the pooled analysis of both sample processing methods, overall sensitivity of cobas MTB was 95% (95% CI, 93%–96%) (Figure 3). Among participants tested with both cobas MTB and Xpert (Moldova and India), sensitivity was 96% (95% CI, 94%–97%) for cobas and 95% (95% CI, 93%–97%) for Xpert (Supplemental Table S2). Among participants tested with both cobas MTB and Ultra (South Africa), sensitivity was 88% (95% CI, 81%–92%) for cobas MTB and 89% (95% CI, 83%–93%) for Ultra (Supplemental Table S2). In smear-positive samples, sensitivity of cobas MTB was 99% (95% CI, 97.8%–99.7%), with four false-negative samples (all detected as MTBC positive by the Xpert assays). When retested, three of the four false-negative samples were detected as MTBC positive by cobas MTB. Head-to-head comparison of cobas MTB to Ultra in South Africa showed similar estimates [98.1% (95% CI, 93.2%–99.5%) versus 100% (95% CI, 96.4%–100%)] (Supplemental Table S2). In smear-negative samples, sensitivity of cobas MTB was 75% (95% CI, 66%–83%). From the 24 cobas MTB false-negative samples, 8 were detected by Xpert or Ultra (semiquantitative: very low: *n* = 5; trace: *n* = 3). Among participants tested with both cobas MTB and Xpert, sensitivity was 80% (95% CI, 70%–88%) for cobas MTB compared with 72% (95% CI, 61%–80%) for Xpert (Supplemental Table S2). Despite the low sensitivity, head-to-head sensitivity estimates for cobas MTB and Ultra in South Africa were comparable [55% (95% CI, 38%–70%) versus 55% (95% CI, 38%–70%)] (Supplemental Table S2).

Specificity of cobas MTB in the pooled analysis of both sample processing methods was 96% (95% CI, 95%–97%), compared with 98% for both Xpert and Ultra (Figure 3). Of 31 false-positive samples on cobas MTB, 9 were also false positive on Xpert or Ultra. Of the 22 cobas MTB false positives that were negative on Xpert or Ultra, all were culture negative on the second sample from the same participant. A total of 17 were followed up, of whom 3 received no treatment and recovered, 2 started treatment and recovered, and 12 did not start treatment and continued to experience symptoms.

**Supplemental Figure S1:**
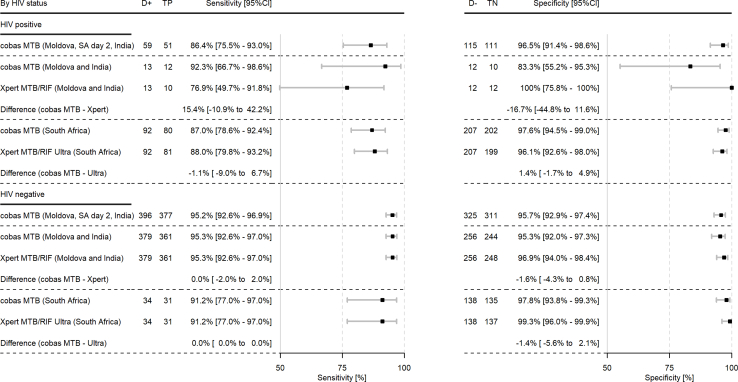
Sensitivity and specificity of cobas MTB, Xpert, and Ultra for *Mycobacterium tuberculosis* complex detection by HIV status. D+, reference standard positive; D−, reference standard negative; SA, South Africa; TN, true negative; TP, true positive.

**Supplemental Figure S2:**
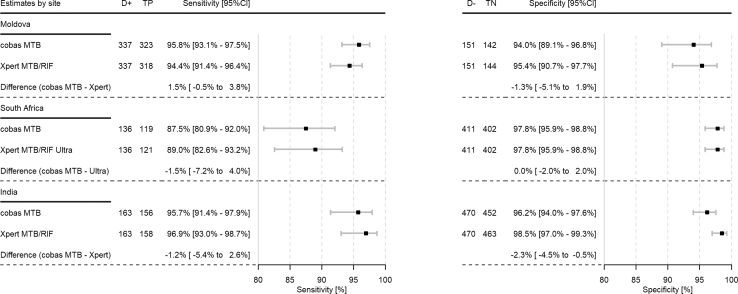
Sensitivity and specificity of cobas MTB, Xpert, and Ultra for *Mycobacterium tuberculosis* complex detection by recruitment site (pooled analysis of both sample processing methods). D+, reference standard positive; D−, reference standard negative; TN, true negative; TP, true positive.

In HIV-positive participants, overall sensitivity of cobas MTB was 86% (95% CI, 75%–93%) and specificity was 97% (95% CI, 91%–99%) (Supplemental Figure S1). Point estimate of sensitivity was similar to that of Xpert Ultra in South Africa (87% versus 88%), whereas comparison to Xpert is not possible because of the small sample size of HIV-positive individuals in Moldova and India. Across recruitment sites, sensitivity and specificity estimates for cobas MTB were similar for Moldova and India [sensitivity of 96% at both sites; and specificity of 94% (95% CI, 89%–97%) and 96% (95% CI, 94%–98%), respectively], with lower sensitivity and higher specificity in South Africa [88% (95% CI, 81%–92%) and 98% (95% CI, 96%–99%), respectively] (Supplemental Figure S2).

**Supplemental Figure S3:**
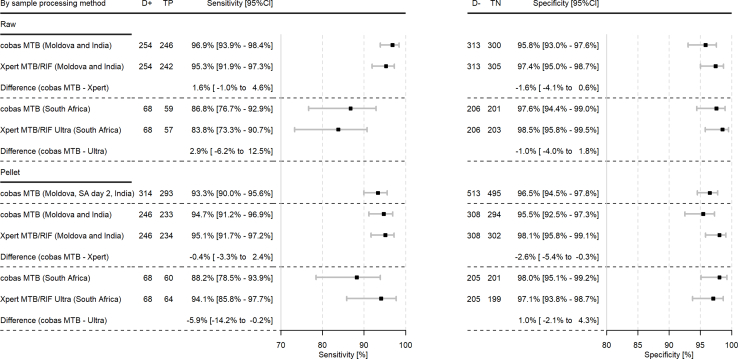
Sensitivity and specificity of cobas MTB, Xpert, and Ultra for *Mycobacterium tuberculosis* complex detection by sample processing method. D+, reference standard positive; D−, reference standard negative; SA, South Africa; TN, true negative; TP, true positive.

Comparable performance was seen when cobas MTB testing was performed on raw and decontaminated pellets, irrespective of the testing site (Supplemental Figure S3): 97% (95% CI, 94%–98%) versus 93% (95% CI, 90%–96%) for Moldova and India and 87% (95% CI, 77%–93%) versus 88% (95% CI, 79%–94%) for South Africa.

**Supplemental Figure S4:**
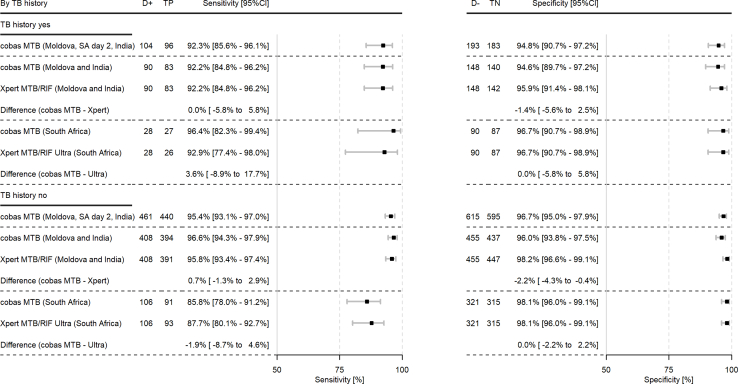
Sensitivity and specificity of cobas MTB, Xpert, and Ultra for *Mycobacterium tuberculosis* complex detection by history of tuberculosis (TB). D+, reference standard positive; D−, reference standard negative; SA, South Africa; TN, true negative; TP, true positive.

Sensitivity of cobas MTB was 95% (95% CI, 93%–97%) in participants with no history of TB and 92% (95% CI, 86%–96%) in participants with a history of TB (Supplemental Figure S4).

### Diagnostic Accuracy of cobas MTB-RIF/INH for Detection of RIF and INH Resistance

**Figure 4:**
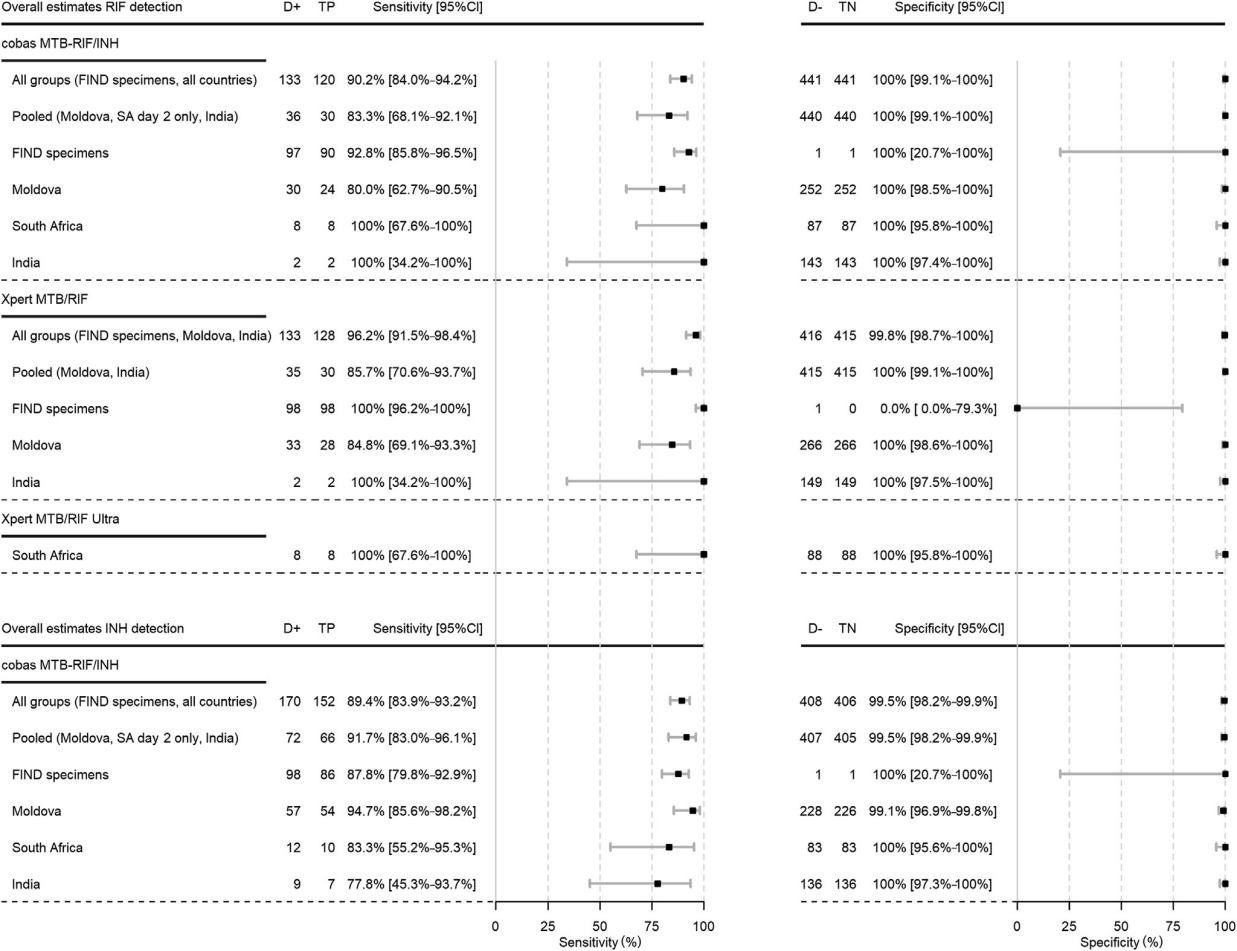
Sensitivity and specificity of cobas MTB-RIF/INH, Xpert, and Ultra for detection of rifampicin (RIF) and isoniazid (INH) resistance (pooled analysis of both sample processing methods). Overall estimates for cobas MTB-RIF/INH include results from both sputa collected in Moldova and India, and only results from the sputum 3 (indirect testing) collected on day 2 in South Africa (SA). Results from sputum 2 (direct testing) collected on day 1 were excluded as no culture reference standard was done on day 1. Overall estimates for Xpert MTB/RIF include results from both sputa collected in Moldova and India. Overall estimates for Xpert MTB/RIF Ultra include results from sputum 2 and 3 collected in South Africa. D+, reference standard positive; D−, reference standard negative; TN, true negative; TP, true positive.

In the pooled analysis of both sample processing methods, sensitivity of cobas MTB-RIF/INH for RIF resistance detection was 90% (95% CI, 84%–94%), compared with 96% (95% CI, 92%–98%) and 100% (95% CI, 68%–100%) for Xpert and Ultra, respectively (Figure 4), with cobas MTB-RIF/INH failing to detect RIF resistance in 13 of 133 samples. Of these samples, 12 had mutations in the *rpoB* gene and 1 was resistant by pDST, but no mutations were detected by WGS (Supplemental Table S3). All seven cobas MTB-RIF/INH false-negative specimens from the FIND Specimen Bank had mutations in the *rpoB* gene; four are listed in the WHO catalog as associated with RIF resistance, and three are listed as associated with resistance-interim, where interim refers to a category where some uncertainty remains that could change with new data. Five of the six false-negative specimens from Moldova had the *rpoB* S450L mutation, whereas the remaining sample was resistant by pDST and wild type by WGS. WGS showed 100% coverage of the *rpoB* gene for this sample, and no large deletions that could explain the discrepancy between pDST and WGS. Four of the six samples were Xpert RIF susceptible, perhaps indicating underlying resistant populations selected during culturing that were missed by the molecular assays when testing was done on the direct specimens. One of six false-negative specimens was detected as resistant when repeated with the cobas MTB RIF/INH assay. Accuracy of cobas MTB-RIF/INH to detect specific *rpoB* mutations was high (Supplemental Table S4). The specificity of cobas MTB-RIF/INH for RIF resistance detection was 100% (95% CI, 99%–100%).

The sensitivity of cobas MTB-RIF/INH for detection of INH resistance in the pooled analysis of both sample processing methods was 89% (95% CI, 84%–93%) (Figure 4). The assay failed to detect INH resistance in 18 of 170 samples. All 12 false-negative specimens from the FIND Specimen Bank were phenotypically resistant to INH and had mutations in either *katG* (mutations at amino acid 315 or elsewhere in the gene) or *fabG1*-*inhA* promoter region (Supplemental Table S3), although some of the mutations are not listed in the WHO mutations catalog. Two of the three false-negative specimens from Moldova had mutations in *katG* (S315T) and *fabG1*-*inhA* (−15 C/T) promoter. The remaining sample was phenotypically resistant but wild type for *katG* and *fabG1*-*inhA* promoter. Retesting of this sample by cobas showed a resistant result. Both false-negative specimens from South Africa were wild type in the targets assessed by WGS, but phenotypically resistant. WGS showed 100% coverage of the *katG* gene, with no large deletions that can explain the discrepant result. The two samples were from the same participant. Retesting of the MGIT DST showed a susceptible result. Both false-negative specimens from India had the −15 C/T mutation in the *inhA* promoter region. The two samples were from the same participant. Accuracy of cobas MTB-RIF/INH to detect specific *katG* and *fabG1-inhA* promoter mutations was high (Supplemental Table S4). Specificity of cobas MTB-RIF/INH for detection of INH resistance was 99.5% (95% CI, 98%–100%). Two false-positive samples (both from the same participant in Moldova) were INH sensitive when retested with cobas MTB-RIF/INH.

### Unsuccessful Rates for cobas MTB and cobas MTB-RIF/INH

The rate of unsuccessful results for cobas MTB was 0.4% (4 of 1057) for raw sputum samples and 0.6% (6 of 1039) for decontaminated pellets (Table 2). Unsuccessful rates for cobas MTB-RIF/INH in raw sputum and decontaminated pellets were 13% (49 of 378) and 9% (33 of 366), respectively, for RIF resistance and 12% (45 of 378) and 8% (29 of 366), respectively, for INH resistance (Table 3). Of 49 and 33 invalid RIF resistance results from raw sputum and decontaminated pellets, 43 and 25 were smear negative, respectively, suggesting low bacillary load. Of 45 and 29 invalid INH resistance results, 39 and 21 were smear negative, respectively. Invalid rates were similar between sites for raw sputum and higher in India for decontaminated pellets.

**Table 2:** Number and Rate of Unsuccessful Results with cobas MTB by Sample Processing Method and Recruitment Site

Variable	All countries	Moldova	South Africa	India
Raw sputum				
Total enrolled (ITT population)	1064	350	373	341
Total tested with cobas MTB	1057	347	369	341
Valid cobas MTB result	1053	346	369	338
Invalid cobas MTB result	4	1	0	3
Invalid rate, %	0.4	0.3	0.0	0.9
NALC-NaOH decontaminated pellet				
Total enrolled (ITT population)	1064	350	373	341
Total tested with cobas MTB	1039	348	350	341
Valid cobas MTB result	1033	348	350	335
Invalid cobas MTB result	6	0	0	6
Invalid rate, %	0.6	0.0	0.0	1.8

ITT, intention to treat; NALC-NaOH, N-acetyl-l-cysteine/sodium hydroxide.

**Table 3:** Number and Rate of Unsuccessful Results with cobas MTB-RIF/INH by Sample Processing Method and Recruitment Site

Variable	All countries	Moldova	South Africa	India	FIND specimens
Raw sputum					
cobas MTB positive (ITT population)	382	214	74	94	100
Total tested with RIF/INH	378	212	74	92	100
Valid RIF result	329	184	64	81	99
Invalid RIF result	49	28	10	11	1
Indeterminate RIF rate, %	13.0	13.2	13.5	12.0	1.0
Valid INH result	333	188	64	81	100
Invalid INH result	45	24	10	11	0
Indeterminate INH rate, %	11.9	11.3	13.5	12.0	0.0
NALC-NaOH decontaminated pellet					
cobas MTB positive (ITT population)	366	198	68	100	
Total tested with RIF/INH	366	198	68	100	
Valid RIF result	333	187	62	84	
Invalid RIF result	33	11	6	16	
Indeterminate RIF rate, %	9.0	5.6	8.8	16.0	
Valid INH result	337	189	62	86	
Invalid INH result	29	9	6	14	
Indeterminate INH rate, %	7.9	4.5	8.8	14.0	

INH, isoniazid; ITT, intention to treat; NALC-NaOH, N-acetyl-l-cysteine/sodium hydroxide; RIF, rifampicin.

## Discussion

This was the first large multicenter prospective diagnostic accuracy study for cobas MTB and cobas MTB-RIF/INH. cobas MTB demonstrated comparable performance to Xpert and Ultra in a diverse clinical population, with a low rate of unsuccessful results. The assay met WHO target product profile sensitivity and specificity criteria for diagnosis of active TB,^9^^,^^10^ and results were generally consistent with previous studies.^4^^,^^5^ cobas MTB-RIF/INH demonstrated high accuracy for RIF and INH resistance detection, similar to a previous report,^5^ with invalid rates ranging from 5% to 16%, dependent on sample type and setting. This suggests that the cobas assays could be suitable alternatives to Xpert and Ultra, with the added benefit of INH resistance detection and the possibility for high-throughput detection.

Consistent with previous reports, sensitivity of cobas MTB and the Xpert assays was lowest in smear-negative samples,^4^^,^^5^^,^^11^, ^12^, ^13^, ^14^ although it remained above the WHO criterion of 60%. The sensitivity of cobas MTB and both Xpert assays decreased in HIV-positive participants compared with HIV-negative participants, likely because of lower bacillary loads in the sputum of individuals living with HIV. In a previous study in South Africa, sensitivity of cobas MTB was unaffected by HIV coinfection.^4^ The reasons for the discrepancy between studies is unclear, but could be related to demographic differences (eg, CD4^+^ cell count or antiretroviral therapy use) or the specific testing protocol used (eg, volume of sample tested). Regardless, sensitivity of cobas MTB in HIV-positive participants remained >86%, comparable to Ultra. High sensitivity in HIV-positive populations is particularly important given the high proportion of TB cases co-infected with HIV in settings such as southern Africa.^1^

Head-to-head comparison of Ultra to cobas MTB showed near perfect equivalence in the samples tested in South Africa. The difference in performance between Xpert and cobas MTB in smear-negative samples (from Moldova and India) may be attributed to cobas MTB targeting multigene targets for MTBC detection, whereas Xpert only targets a single-copy gene. In addition, the differences in specificity estimates may be attributed to the ability of cobas MTB to detect free DNA from dead bacilli for which detection by Xpert is limited. Ultra and Xpert performance estimates cannot be directly compared as the tests were not done on the same patients, were evaluated in different settings with different TB populations (eg, South Africa has a high TB and HIV co-infection rate), and used different sample flows and testing volumes. The low sensitivity (55%) for both Ultra and cobas MTB in smear-negative specimens from South Africa should be interpreted with caution as estimates are imprecise because of the small sample size. Sensitivity of cobas MTB was higher in Moldova and India, demonstrating how differences in study populations can impact assay performance. This emphasizes the need for multicountry diagnostic accuracy studies using WHO-recommended assays as comparator tests.

The sensitivity of cobas MTB-RIF/INH for RIF resistance detection was 90%, compared with 96% and 100% for Xpert and Ultra, respectively. The number of discrepant results may have been inflated as the analysis included index assay results from both sample types (raw and processed), whereas WGS and DST were only performed on one culture-positive sample per participant. One false-negative sample was resistant when retested with cobas MTB-RIF/INH, whereas for four of the six discrepant samples from Moldova, Xpert also failed to detect RIF resistance. This may indicate the presence of mixed bacterial subpopulations with a selection bias during culturing that were missed by direct testing of the molecular assays.

The sensitivity of cobas MTB-RIF/INH for INH resistance detection was also high despite the inclusion of samples from the FIND Specimen Bank, with rare *katG* mutations not covered by the cobas MTB-RIF/INH assay. There is an urgent need for improved diagnostics to detect INH resistance, as individuals diagnosed with INH-resistant TB require a modification of the standard treatment regimen. In addition, failure to detect INH resistance combined with the existence of rare RIF resistance mutations not detectable by commercially available molecular assays leads to the spread and expansion of multi–drug-resistant TB clones.^15^

The high unsuccessful rates for cobas MTB-RIF/INH are a consequence of the assay's lower limit of detection for single-copy targets compared with cobas MTB, which targets multicopy genetic elements. Most nondeterminate cobas MTB-RIF/INH samples were smear negative.

Limitations in this study include the differences in sample flow and comparator test for South Africa versus Moldova and India. Additionally, pDST was done using the WHO-recommended RIF critical concentration of 1 μg/mL at the beginning of the study. However, after study initiation, new WHO guidelines were released, recommending a critical concentration of 0.5 μg/mL for RIF.^16^ Therefore, in this study, pDST may have missed any samples with *rpoB* mutations conferring minimal inhibitory concentrations between 0.5 and 1 μg/mL. These samples would still have been considered as resistant if the mutation was detected by WGS; thus, the impact on study results is likely to have been limited. A large proportion of resistant samples used in this study to evaluate the performance of the cobas MTB-RIF/INH assay to detect RIF and INH resistance were selected from the FIND Specimen Bank. Although the results from this study do not suggest that these samples showed reduced integrity because of long-term storage, there may have been a selection bias in the specific *rpoB* and *katG* mutations that were represented in this study.

In summary, cobas MTB and cobas MTB-RIF/INH demonstrated high diagnostic performance in a diverse clinical population. These assays may be suitable alternatives to existing molecular tests for the detection of TB and drug resistance, particularly in settings where high-throughput testing is required.

## Disclosure Statement

M.d.V., A.P.-N., A.M., S.O., P.N., and M.R. are employees of FIND. S.G.S., J.M., I.K., and A.P. were employed by FIND at the time of the study. S.S., S.S.C., and K.D. are employees of FIND, India. F.P.M. changed employment from Research Center Borstel (Borstel, Germany) to Roche (Rotkreuz, Switzerland) after the completion of the study in July 2022. (A.M., S.O., P.N., S.S., S.S.C., J.M., I.K., and A.P. are Study Group members.)
