# Supplementary material for: Accuracy of cobas MTB and MTB-RIF/INH for Detection of Mycobacterium tuberculosis and Drug Resistance
Source: J Mol Diagn. 2024 Aug;26(8):708–18. doi: 10.1016/j.jmoldx.2024.05.004 (PMC11298579; doi:10.1016/j.jmoldx.2024.05.004)
Supplement: Supplemental Table S1 [file mmc1.docx]

**Supplemental Table S1.** Participant enrollment and exclusions overall and by recruitment site

|  | **All** | **Moldova** | **South Africa** | **India** |
| --- | --- | --- | --- | --- |
| ***Total Enrolled*** | 1064 | 350 | 373 | 341 |
| Eligible | 968 | 293 | 337 | 338 |
| Not Eligible | 96 | 57 | 36 | 3 |
| Symptoms and duration | 92 | 54 | 36 | 2 |
| TB treatment in the last 6 months | 4 | 3 | 0 | 1 |
| Participant discontinuation/withdrawal* | 24 | 0 | 24 | 0 |
| Early exclusion† | 2 | 2 | 0 | 0 |
| ***Total excluded*** | 122 | 59 | 60 | 3 |
| ***Total eligible for analysis*** | 942 | 291 | 313 | 338 |
| Culture contaminated | 73 | 35 | 37 | 1 |
| Data entry error | 1 | 1 | 0 | 0 |
| No valid cobas result | 10 | 1 | 1 | 8 |
| No Xpert or Ultra result | 9 | 8 | 1 | 0 |
| **Total Analysed PP** | 849 | 246 | 274 | 329 |

*Withdrawals can also include participants who failed to provide the correct volume and number of samples
†Due to sample mix-up
mITT, modified intention-to-treat population; PP, per protocol population
