# Supplementary material for: Accuracy of cobas MTB and MTB-RIF/INH for Detection of Mycobacterium tuberculosis and Drug Resistance
Source: J Mol Diagn. 2024 Aug;26(8):708–18. doi: 10.1016/j.jmoldx.2024.05.004 (PMC11298579; doi:10.1016/j.jmoldx.2024.05.004)
Supplement: Supplemental Table S2 [file mmc2.docx]

**Supplemental Table S2.** Head-to-head performance comparison of cobas MTB and Xpert MTB/RIF or Xpert Ultra for MTBC detection

| **Assay** | **N** | **TP** | **FN** | **TN** | **FP** | **Sensitivity, % (95% CI)** | **Specificity, % (95% CI)** |
| --- | --- | --- | --- | --- | --- | --- | --- |
| ***All samples*** |  |  |  |  |  |  |  |
| cobas MTB (Moldova, SA day 2, India) | 1394 | 539 | 29 | 795 | 31 | 94.9 (92.8–96.4) | 96.2 (94.7–97.3) |
| cobas MTB (pooled Moldova and India) | 1121 | 479 | 21 | 594 | 24 | 95.8 (93.7–97.2) | 95.8 (93.7–97.0) |
| Xpert MTB/RIF (pooled Moldova and India) | 1121 | 476 | 24 | 607 | 14 | 95.2 (93.0–96.8) | 97.7 (96.3–98.7) |
| Difference (cobas MTB* – Xpert MTB/RIF) |  |  |  |  |  | +0.6 (−1.2 to 2.5) | -2.1 (−3.9 to 0.6) |
| cobas MTB (South Africa) | 547 | 119 | 17 | 402 | 9 | 87.5 (80.9–92.0) | 97.6 (95.6–98.7) |
| Xpert Ultra (South Africa) | 547 | 121 | 15 | 402 | 9 | 89.0 (82.6–93.2) | 97.6 (95.6–98.7) |
| Difference (cobas MTB† – Xpert Ultra) |  |  |  |  |  | −1.5 (−7.2 to 4.0) | +0.0 (−2.0 to 2.0) |
| ***Smear positive samples*** |  |  |  |  |  |  |  |
| cobas MTB (Moldova, SA day 2, India) | 470 | 466 | 4 | 0 | 0 | 99.1 (97.8–99.7) |  |
| cobas MTB (pooled Moldova and India) | 418 | 414 | 4 | 0 | 0 | 99.0 (97.6–99.6) |  |
| Xpert MTB/RIF (pooled Moldova and India) | 418 | 418 | 0 | 0 | 0 | 100 (99.1–100) |  |
| Difference (cobas MTB* – Xpert MTB/RIF) |  |  |  |  |  | −1.0 (−2.4% to −0.0) |  |
| cobas MTB (South Africa) | 103 | 101 | 2 | 0 | 0 | 98.1 (93.2–99.5) |  |
| Xpert Ultra (South Africa) | 103 | 103 | 0 | 0 | 0 | 100 (96.4–100) |  |
| Difference (cobas MTB† – Xpert Ultra) |  |  |  |  |  | −1.9 (−6.8 to −1.7) | +0.0 (0.0 to 0.0) |
| ***Smear negative samples*** |  |  |  |  |  |  |  |
| cobas MTB (Moldova, SA day 2, India) | 923 | 73 | 24 | 795 | 31 | 75.3 (65.8–82.8) | 96.2 (94.7–97.3) |
| cobas MTB (pooled Moldova and India) | 702 | 65 | 16 | 594 | 27 | 80.2 (70.3–87.5) | 95.7 (93.7–97.0) |
| Xpert MTB/RIF (pooled Moldova and India) | 702 | 58 | 23 | 607 | 14 | 71.6 (61.0–80.3) | 97.7 (96.3–98.7) |
| Difference (cobas MTB* – Xpert MTB/RIF) |  |  |  |  |  | +8.6 (−0.8 to 18.8) | −2.1 (−3.9 to −0.6) |
| cobas MTB (South Africa) | 444 | 18 | 15 | 402 | 9 | 54.5 (38.0–70.2) | 97.8 (95.9–98.8) |
| Xpert Ultra (South Africa) | 444 | 18 | 15 | 402 | 9 | 54.5 (38.0–70.2) | 97.8 (95.9–98.8) |
| Difference (cobas MTB† – Xpert Ultra) |  |  |  |  |  | +0.0 (−19.8 to 19.8) | +0.0 (−2.0 to 2.0) |

CI, confidence interval; FN, false negative; FP, false positive; MTBC, *Mycobacterium tuberculosis* complex; SA, South Africa; TB, tuberculosis; TN, true negative; TP, true positive; * data from Moldova and India only; † data from SA only
