# Supplementary material for: Accuracy of cobas MTB and MTB-RIF/INH for Detection of Mycobacterium tuberculosis and Drug Resistance
Source: J Mol Diagn. 2024 Aug;26(8):708–18. doi: 10.1016/j.jmoldx.2024.05.004 (PMC11298579; doi:10.1016/j.jmoldx.2024.05.004)
Supplement: Supplemental Table S3 [file mmc3.docx]

**Supplemental Table S3.** Characteristics of discrepant samples as detected by cobas MTB-RIF/INH when using pDST and WGS as composite reference standard

| **Discrepancy** | **Participant ID** | **Country** | **Matrix** | **pDST** | **WGS** | **cobas MTB-RIF/INH** | **Xpert** | **cobas MTB-RIF/ INH repeat** | **pDST repeat** | **WHO catalogue classification V2 [6]** | **Listed as detectable in cobas MTB-RIF/INH IFU** |
| --- | --- | --- | --- | --- | --- | --- | --- | --- | --- | --- | --- |
| **RIF resistance** |  |  |  |  |  |  |  |  |  |  |  |
| False negative | TB024010028 | Moldova | Sputum | Susceptible | S450L | Susceptible | Susceptible | Not done | Not done | Assoc. with resistance | Listed |
| False negative |  |  | Pellet | Susceptible | S450L | Susceptible | Susceptible | Not done | Not done | Assoc. with resistance | Listed |
| False negative | TB024010116 | Moldova | Sputum | Resistant | S450L | Susceptible | Resistant | Not done | Not done | Assoc. with resistance | Listed |
| False negative | TB024010192 | Moldova | Sputum | Resistant | S450L | Susceptible | Resistant | Resistant | Not done | Assoc. with resistance | Listed |
| False negative | TB024010199 | Moldova | Sputum | Susceptible | S450L | Susceptible | Susceptible | Not done | Not done | Assoc. with resistance | Listed |
| False negative | TB024010267 | Moldova | Pellet | Resistant | WT | Susceptible | Susceptible | Susceptible | Not done | — | — |
| False negative | F-01-67-0033 | FIND specimen | Sputum | Resistant | D435V | Susceptible | Resistant | Not done | Not done | Assoc. with resistance | Listed |
| False negative | 14-13767 | FIND specimen | Sputum | Susceptible | D435G, M434I | Susceptible | Resistant | Not done | Not done | M434I and D435G assoc. with resistance-interim | D435G listed; M434I and P454 not listed |
| False negative | 15-01839 | FIND specimen | Sputum | Resistant | H445P | Susceptible | Resistant | Not done | Not done | Assoc. with resistance-interim | Not listed |
| False negative | 15-01850 | FIND specimen | Sputum | Resistant | Deletion | Susceptible | Resistant | Not done | Not done | In frame deletion, not listed | Not listed |
| False negative | 15-16889 | FIND specimen | Sputum | Susceptible | D435Y | Susceptible | Resistant | Not done | Not done | Assoc. with resistance | Listed |
| False negative | 17-8411 | FIND specimen | Sputum | Resistant | L452P, N437D, T444A | Susceptible | Resistant | Not done | Not done | L452P assoc. with resistance; N437D assoc. with resistance-interim; T444A not listed | L452P listed; N437D and T444A not listed |
| False negative | F-4-003 | FIND specimen | Sputum | Susceptible | D435Y, P457T | Susceptible | Resistant | Not done | Not done | D435Y assoc. with resistance; P454T not listed | D435Y listed; P454T not listed |
| **INH resistance** |  |  |  |  |  |  |  |  |  |  |  |
| False negative | TB024010028 | Moldova | Sputum | Susceptible | S315T, -15 C/T | Susceptible | N/A | Not done | Not done | S315T and-15 C/T assoc. with resistance | S315T and -15 C/T listed |
| False negative |  |  | Pellet | Susceptible | S315T, -15 C/T | Susceptible | N/A | Not done | Not done | S315T and-15 C/T assoc. with resistance | S315T and -15 C/T listed |
| False negative | TB024010267 | Moldova | Pellet | Resistant | WT | Susceptible | N/A | Resistant | Not done | — | — |
| False negative | TB024030358 | South Africa | Sputum | Resistant | WT | Susceptible | N/A | Susceptible | Sensitive | — | — |
| False negative |  |  | Pellet | Resistant | WT | Susceptible | N/A | Susceptible | Sensitive | — | — |
| False negative | TB024020296 | India | Sputum | Susceptible | -15 C/T | Susceptible | N/A | Not done | Not done | Assoc. with resistance | Listed |
| False negative |  |  | Pellet | Susceptible | -15 C/T | Susceptible | N/A | Not done | Not done | Assoc. with resistance | Listed |
| False negative | F-01-67-0024 | FIND specimen | Sputum | Resistant | -15 C/T | Susceptible | N/A | Not done | Not done | Assoc. with resistance | Listed |
| False negative | 15-01779 | FIND specimen | Sputum | Resistant | S315G | Susceptible | N/A | Not done | Not done | Not listed | Not listed |
| False negative | 15-01784 | FIND specimen | Sputum | Resistant | S315N | Susceptible | N/A | Not done | Not done | Assoc. with resistance | Listed |
| False negative | 15-01787 | FIND specimen | Sputum | Resistant | W191R | Susceptible | N/A | Not done | Not done | Not listed | Not listed |
| False negative | 15-01795 | FIND specimen | Sputum | Resistant | E195E | Susceptible | N/A | Not done | Not done | Not listed | Not listed |
| False negative | 15-01806 | FIND specimen | Sputum | Resistant | P325L, P325A | Susceptible | N/A | Not done | Not done | Not listed | Not listed |
| False negative | 15-01810 | FIND specimen | Sputum | Resistant | S383A, Y337S | Susceptible | N/A | Not done | Not done | Not listed | Not listed |
| False negative | 15-01850 | FIND specimen | Sputum | Resistant | S315N, I317V | Susceptible | N/A | Not done | Not done | S315N assoc. with resistance; I317V not listed | S315N listed; I317V not listed |
| False negative | 15-08892 | FIND specimen | Sputum | Resistant | S315G | Susceptible | N/A | Not done | Not done | Not listed | Not listed |
| False negative | 16-04673 | FIND specimen | Sputum | Resistant | -15 C/T | Susceptible | N/A | Not done | Not done | Assoc. with resistance | Listed |
| False negative | F-063S113 | FIND specimen | Sputum | Resistant | S315T | Susceptible | N/A | Not done | Not done | Assoc. with resistance | Listed |
| False negative | F-4-001 | FIND specimen | Sputum | Resistant | -17 G/T | Susceptible | N/A | Not done | Not done | Assoc. with resistance-interim | Not listed |
| False positive | TB024010338 | Moldova | Sputum | Susceptible | WT | Resistant | N/A | Susceptible | Susceptible | — | — |
| False positive |  |  | Pellet | Susceptible | WT | Resistant | N/A | Susceptible | Susceptible | — | — |

Assoc., associated; IFU, instructions for use; pDST, phenotypic drug susceptibility testing; WGS, whole genome sequencing
