# Supplementary material for: Accuracy of cobas MTB and MTB-RIF/INH for Detection of Mycobacterium tuberculosis and Drug Resistance
Source: J Mol Diagn. 2024 Aug;26(8):708–18. doi: 10.1016/j.jmoldx.2024.05.004 (PMC11298579; doi:10.1016/j.jmoldx.2024.05.004)
Supplement: Supplemental Table S4 [file mmc4.docx]

**Supplemental Table S4.** Accuracy of cobas MTB-RIF/INH for the detection of specific *rpoB* mutations

| ***rpoB* mutation** | **Number of  samples tested** | **Number detected by cobas MTB-RIF/INH** | **Percentage detected by cobas MTB-RIF/INH** |
| --- | --- | --- | --- |
| 761102i3 TTC | 1 | 1 | 100 |
| D435G (gac/gGc);I491F (atc/Ttc) | 1 | 1 | 100 |
| D435G (gac/gGc);L452P (ctg/cCg) | 1 | 1 | 100 |
| D435V (gac/gTc) | 7 | 6 | 86 |
| D435Y (gac/Tac) | 4 | 2 | 50 |
| H445D (cac/Gac) | 7 | 7 | 100 |
| H445D (cac/Gac);H445R (cac/cGc) | 1 | 1 | 100 |
| H445L (cac/cTc) | 3 | 3 | 100 |
| H445N (cac/Aac) | 2 | 2 | 100 |
| H445P (cac/cCc) | 1 | 0 | 0 |
| H445R (cac/cGc) | 2 | 2 | 100 |
| H445Y (cac/Tac) | 8 | 8 | 100 |
| L430P (ctg/cCg) | 6 | 6 | 100 |
| L430P (ctg/cCg);D435G (gac/gGc) | 2 | 2 | 100 |
| L452P (ctg/cCg) | 6 | 6 | 100 |
| L452P (ctg/cCg); N437D (aac/Gac);T444A (acc/Gcc) | 1 | 0 | 0 |
| M434I (atg/atA);D435G (gac/gGc) | 1 | 0 | 0 |
| Q429H (cag/caC);H445R (cac/cGc | 1 | 1 | 100 |
| Q432K (caa/Aaa) | 2 | 2 | 100 |
| Q432L (caa/cTa);K446E (aag/Gag) | 1 | 1 | 100 |
| Q432P (caa/cCa) | 1 | 1 | 100 |
| S441L (tcg/tTg) | 1 | 1 | 100 |
| S450L (tcg/tTg) | 64 | 59 | 92 |
| S450W (tcg/tGg) | 6 | 6 | 100 |
| S450L (tcg/tTg);S450S (tcg/tcC) | 1 | 1 | 100 |
| T400A (acc/Gcc);S450L (tcg/tTg) | 1 | 1 | 100 |
| ***k*atG mutation** | **Number of  samples tested** | **Number detected by cobas MTB-RIF/INH** | **Percentage detected by cobas MTB-RIF/INH** |
| E195E | 1 | 0 | 0% |
| P325L, P325A | 1 | 0 | 0% |
| S315G | 2 | 0 | 0% |
| S315N | 4 | 4 | 100% |
| S315N, I317V | 2 | 0 | 0% |
| S315T | 134 | 131 | 98% |
| S383A, Y337S | 1 | 0 | 0% |
| W191R | 1 | 0 | 0% |
| ***inhA* promoter mutation** | **Number of  samples tested** | **Number detected by cobas MTB-RIF/INH** | **Percentage detected by cobas MTB-RIF/INH** |
| -15 C/T | 64 | 58 | 91% |
| -17 G/T | 2 | 1 | 50% |
| -8 T/A | 3 | 3 | 100% |
| -8 T/C | 2 | 2 | 100% |
